# Random or predictable?: Adoption patterns of chronic care management practices in physician organizations

**DOI:** 10.1186/s13012-017-0639-z

**Published:** 2017-08-24

**Authors:** Isomi M. Miake-Lye, Emmeline Chuang, Hector P. Rodriguez, Gerald F. Kominski, Elizabeth M. Yano, Stephen M. Shortell

**Affiliations:** 1 Department of Health Policy and Management, UCLA Jonathan and Karin Fielding School of Public Health, 640 Charles E. Young Drive South, Los Angeles, CA 90024 USA; 2 Center for the Study of Healthcare Innovation, Implementation & Policy (CSHIIP), VA Greater Los Angeles Healthcare System, 11301 Wilshire Boulevard, Los Angeles, CA 90073 USA; 3 Department of Health Policy and Management, UC-Berkeley School of Public Health, 50 University Hall, Berkeley, CA 94720 USA

**Keywords:** Chronic care, Care management practices, Adoption, Physician organizations

## Abstract

**Background:**

Theories, models, and frameworks used by implementation science, including Diffusion of Innovations, tend to focus on the adoption of one innovation, when often organizations may be facing multiple simultaneous adoption decisions. For instance, despite evidence that care management practices (CMPs) are helpful in managing chronic illness, there is still uneven adoption by physician organizations. This exploratory paper leverages this natural variation in uptake to describe inter-organizational patterns in adoption of CMPs and to better understand how adoption choices may be related to one another.

**Methods:**

We assessed a cross section of national survey data from physician organizations reporting on the use of 20 CMPs (5 each for asthma, congestive heart failure, depression, and diabetes). Item response theory was used to explore patterns in adoption, first considering all 20 CMPs together and then by subsets according to disease focus or CMP type (e.g., registries, patient reminders). Mokken scale analysis explored whether adoption choices were linked by disease focus or CMP type and whether a consistent ordering of adoption choices was present.

**Results:**

The Mokken scale for all 20 CMPs demonstrated medium scalability (*H* = 0.43), but no consistent ordering. Scales for subsets of CMPs sharing a disease focus had medium scalability (0.4 < *H* < 0.5), while subsets sharing a CMP type had strong scalability (*H* > 0.5). Scales for CMP type consistently ranked diabetes CMPs as most adoptable and depression CMPs as least adoptable. Within disease focus scales, patient reminders were ranked as the most adoptable CMP, while clinician feedback and patient education were ranked the least adoptable.

**Conclusions:**

Patterns of adoption indicate that innovation characteristics may influence adoption. CMP dissemination efforts may be strengthened by encouraging traditionally non-adopting organizations to focus on more adoptable practices first and then describing a pathway for the adoption of subsequent CMPs. Clarifying why certain CMPs are “less adoptable” may also provide insights into how to overcome CMP adoption constraints.

**Electronic supplementary material:**

The online version of this article (doi:10.1186/s13012-017-0639-z) contains supplementary material, which is available to authorized users.

## Background

Many theories, models, and frameworks used to describe adoption focus on the adoption of a single innovation [1–7], e.g., to identify innovation features affecting adoption or to explain why one particular innovation was chosen over another. Less well understood is the role that differences between innovations might play when multiple adoption decisions are happening simultaneously.

Multiple frameworks, including Rogers’s Diffusion of Innovations, posit that characteristics of the innovations can affect uptake [5]. Key characteristics identified in the broader literature as affecting adoption of an innovation—defined by Rogers as any idea, practice, or object that is perceived as new by the adopter—include compatibility, relative advantage, trialability, and observability [7–10]. Compatibility addresses the extent to which an innovation is congruent with a potential adopter’s existing routines, beliefs, and priorities [5, 8]. Relative advantage speaks to the perceived benefits of the innovation relative to current practice or other, alternative innovations. Trialability refers to the ease with which an innovation can be tested, and finally, observability is the speed and ease with which an innovation’s benefits can be perceived by the adopter. Any or all of these factors can facilitate or impede innovation uptake [9].

A major focus in current healthcare reform discussions is how to improve chronic illness care [11, 12]. Chronic illness care is a multi-faceted problem requiring modification of a number health system factors [13–17]. To fully address the complexities of chronic conditions and support productive interactions between patients and practice teams, multiple related innovation adoption decisions may be necessary.

Care management practices (CMPs), which refer to evidence-based guidelines, care management systems, and disease management programs based on shared principles, exemplify the multiple adoption challenges facing many healthcare organizations. Examples of highly recommended CMPs include sending patients reminders for preventive or follow-up care related to their chronic condition, educating patients about their condition, using reminders to alert providers of guideline-concordant care needs at the time of an appointment, providing feedback to providers about their quality of care, and maintaining a registry of patients with a particular chronic condition [13, 15]. These CMPs can be applied to any number of chronic conditions and have consistently been shown to improve quality of care and clinical outcomes for a number of chronic conditions [13, 15, 18]. Organizations deciding whether to adopt CMPs must also determine whether to adopt each CMP individually or in tandem with other CMPs.

Despite this evidence that CMPs are helpful in managing chronic illness, there is still uneven adoption of CMPs by physician organizations [12]. To better understand differential adoption of CMPs, researchers have identified organizational characteristics associated with CMP usage, such as organization size, ownership, and receipt of financial incentives for quality [12, 19]. However, this past work has not systematically analyzed innovation characteristics across CMPs to determine how innovation traits may contribute to differential adoption.

Given the variable uptake of CMPs [12], a better understanding of how innovation characteristics may be linked to adoption is warranted. Several descriptive patterns have emerged that support further inspection: analysis of national physician organization data revealed that in 2006, the average organization had adopted roughly twice as many CMPs for diabetes as for depression [12]. In addition, between 2000 and 2006, the use of disease registries that enabled organizations to identify patients with a particular disease grew faster than other types of CMPs [19]. Differential adoption for subsets of the CMPs, such as the relatively high adoption of diabetes CMPs and disease registries, suggests the choice of which CMP to adopt may be related to certain characteristics of the CMPs in question. To our knowledge, this study is the first to empirically examine the patterns for multiple adoption choices, which reflects the reality shared by many adopter organizations that are making many decisions simultaneously.

Because all CMPs support the type of healthcare reform conceptualized in models such as the Chronic Care Model [17], the choice to adopt any specific CMP may be related to the choice to adopt other CMPs. This reflects the hypothesis that certain organizations may embrace comprehensive health system reform according to Chronic Care Model principles, while others may selectively adopt CMPs based on specific characteristics of interest. In this exploratory study, we also hypothesize that CMPs with similar disease foci or types may share key innovation characteristics that inhibit or encourage their adoption. While disease focus and CMP type are not direct measures of specific innovation characteristics described by the Diffusion of Innovations framework, using these traits to categorize CMPs may allow patterns to become apparent that could be explored further; connections to specific Diffusion of Innovations characteristics could become hypotheses for future work.

If, for instance, an organization is looking to tackle asthma as a key issue, all the CMPs focusing on asthma would be perceived as having high compatibility, and the Diffusion of Innovations framework would suggest that this compatibility would make asthma CMPs more likely to be adopted than CMPs for other chronic conditions [9]. Similarly, the analogous technical expertise required to implement all disease registries might make an organization that adopted a disease registry for diabetes more likely to adopt registries for other chronic conditions.

To understand this milieu of adoption decisions, we used national survey data to examine adoption choices among physician organizations reporting on use of CMPs for four highly prevalent chronic conditions (asthma, CHF, depression, and diabetes). We utilized item response theory techniques to compare and contrast the adoption of multiple CMPs with shared characteristics (e.g., disease focus, CMP type) [20, 21]. Specific study objectives are to describe patterns in adoption of care management practices by physician organizations and to understand better how organizations’ adoption choices are related to one another. Potential extensions of this work to other innovation characteristics and settings are also discussed.

## Methods

### Data source

The third wave of the National Survey of Physician Organizations (2012–2013) was used for the analysis. The National Survey of Physician Organizations is a nationally representative survey of physician organizations caring for patients with chronic conditions including asthma, CHF, depression, and diabetes. These highly prevalent chronic conditions account for a significant percentage of national expenditures [22–26]. Given their contribution to the overall burden of chronic disease in the USA, these diseases have been the foci of many systems’ CMP efforts, resulting in positive outcomes [13, 15, 18]. As a result, the survey focuses on these particular CMPs.

A total of 1398 participant organizations responded, yielding an overall adjusted response rate of 50% [27]. The survey was administered as a 40-min telephone survey or web-based survey to either a lead physician or administrator within each participating organization. Additional information regarding the survey’s methodology is available elsewhere [27, 28].

### Measures

Organizations were separately asked about whether they used five different types of CMPs for four chronic diseases (asthma, CHF, depression, and diabetes). We constructed a total of 20 CMP measures as dichotomous variables denoting the presence or absence of each CMP type for each chronic condition:

#### Patient education

If organizations responded “yes” to “Does your [organizations] have any non-physician staff, for example, nurses, dieticians, or health educators, who have time set aside to meet with and/or call patients to help educate them about managing their [disease]?,” they were considered an adopter of the education CMP type.

#### Provider feedback

If organizations responded “less than half,” “half or more,” or “all” to “Approximately what proportion – if any – of your physicians who care for patients with [disease] receive data from your medical group on the quality of their care for patients with [disease]?,” they were considered an adopter in the feedback CMP type. If organizations responded “none,” they were considered non-adopters.

#### Provider reminders

If organizations responded “less than half,” “half or more,” or “all” to “Please consider the extent, if any, that your group provides physicians with guideline-based reminders – that they see at the time they are seeing the patient – for services the patient should receive. An example would be a pop-up within an electronic medical record or an appropriate reminder attached to the front of the chart each time that they see the patient,” they were considered an adopter in the provider reminder CMP type. If organizations responded “none,” they were considered non-adopters.

#### Patient reminders

If organizations responded “less than half,” “half or more,” or “all” to “To approximately what proportion, if any, of the patients with the following diseases does your [organization] routinely send reminders for preventive or follow-up care [for disease]?,” they were considered an adopter in the patient reminder CMP type. If organizations responded “none,” they were considered non-adopters.

#### Disease registries

If organizations responded “yes” to “For a majority of the patients in your [organization] with [disease]... does your [organization] maintain an electronic registry?” or “does your [organization] maintain a list of patients?,” they were considered an adopter in the registry CMP type.

### Analyses

To describe patterns of CMP adoption, we first examined the extent to which CMP use was correlated. Bivariate analyses explored pairwise correlations within the full set of 20 CMPs [29]. When looking at the matrix of correlation coefficients, we expected that all CMPs would be positively correlated due to common theoretical underpinnings and implementation requirements. Within subsets of CMPs, we expected each CMP to be more strongly correlated with other CMPs sharing a disease focus (e.g., diabetes) or the same type (e.g., patient education) than CMPs that did not share these traits. A correlation coefficient of 0.3 to 0.5 was considered low, 0.5 to 0.7 was considered moderate, 0.7 to 0.9 was considered high, and 0.9 or higher was considered very high [30]. Groups of stronger correlations were useful in indicating the appropriateness of Mokken scale analyses.

Mokken scale analysis is a non-parametric form of item response theory that can be applied to determine whether specific measures function independently or are connected by a shared latent trait [20, 21, 31–33]. An additional advantage of the Mokken scale analysis over classical test theory (e.g., factor analysis) is the ability to determine not only whether measures are related but whether they may be ordered in terms of difficulty [20, 33]. In this context, difficulty refers to whether there may be a hierarchical ordering to responses, i.e., responses to more “difficult” measures only occur in conjunction with responses to “less difficult” measures. For example, two ordered measures might be (1) I have used a computer at least once in the last 30 days, and (2) I have used a computer at least five times in the last 30 days. The measures are considered ordered because a positive response to the second, more difficult item implies a positive response to the first item as well.

In the current study, Mokken scale analyses were applied to determine whether organizations’ CMP adoption decisions occur separately or may be related based on a shared trait such as disease focus or CMP type. An important secondary objective was to assess whether adoption choices may also be ordered. For instance, given the information technology infrastructure required to implement certain CMPs, we might expect that an organization adopting provider feedback for diabetes would also utilize provider reminders for diabetes, but not vice versa. In this scenario, provider feedback would be considered a more difficult—and therefore, less adoptable—measure than provider reminders.

Within each group—the group including all CMPs, the disease focus groups, and the CMP type groups—we explored whether there is consistent ordering in the adoption of the specific CMPs across physician organizations. We refer to the ordering of CMPs in any series as “adoptability.” CMPs that are “more adoptable,” or of a lower rank in a series, will be adopted by all organizations adopting any higher ranked “less adoptable” CMP. The following combinations of CMPs were analyzed: (1) all 20 CMPs considered together in an all-inclusive set; (2) CMPs organized by shared disease focus of asthma, CHF, depression, and diabetes; and (3) CMPs organized according to the five CMP types of patient education, provider feedback, provider reminders, patient reminders, and patient registries.

Sets of CMPs were considered to be scales if they meet three assumptions: unidimensionality for the latent trait, local stochastic independence, and monotonicity [21, 33, 34]. Descriptions and results of specification tests are presented in Additional file 1. In the Mokken scale procedure, items are broken into as many scales as necessary if all items being examined do not meet these assumptions for one unifying scale. Scales with an overall Loevinger’s *H* coefficient of scalability at or above 0.5 were considered strong, at or above 0.4 were considered medium, above 0.3 were considered weak, and 0.3 and lower were not considered to be scales [20]. For our analyses, we compared and contrasted the strength of the scales of CMPs to determine if certain shared traits among CMPs connote stronger relationships than others.

A crucial feature of the Mokken scale procedure is that within groups of CMPs that form a scale, the CMPs can be ordered [33]. To order CMPs by adoptability, the scale must also satisfy the additional assumption necessary to demonstrate consistent ranking of CMPs for all respondent organizations, the non-intersection of the Pmatrix curves (described in Additional file 1) [31]. This second phase of the Mokken scale procedure allowed us to see if there was a ranking or ordering of CMPs in the all-inclusive scale with all 20 CMPs, in scales of CMPs with the same disease focus, or in scales of CMPs using the same CMP type.

We used Stata (version StataSE 14) to conduct our analyses, using the msp and loevh commands presented by Hardouin and colleagues for Mokken analyses [21, 31, 34, 35]. We used the pairwise option for all analyses to retain as much information as possible. All Mokken scale analyses had no missing values (*n* = 1398). For the measures dichotomizing ordinal responses—provider feedback, provider reminders, and patient reminders—sensitivity analyses were run with the “less than half” response included as a non-adopter. This change did not affect our findings.

### Ethics

This study was reviewed and approved by the University of California, Berkeley Committee for the Protection of Human Subjects. The University of California, Los Angeles Office of the Human Research Protection Program agreed to a memorandum of understanding resulting in reliance on the University of California, Berkeley Committee for Protection of Human Subjects for study review and approval.

## Results

**Fig. 1:**
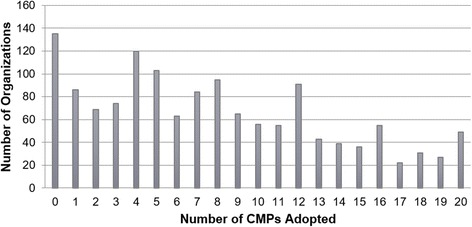
Overall distribution of care management practice adoption by physician organizations (*N* = 1398)

Of the 1398 responding organizations, only 135 (9.7%) had not adopted any care management practices (see Fig. 1). The organizations varied in the number of CMPs they adopted, with an average of 7.84 (standard deviation (SD) = 5.71) adopted CMPs per organization. The majority of organizations had adopted fewer than 8 CMPs, but 49 organizations (3.5%) had adopted all 20 CMPs.

**Fig. 2:**
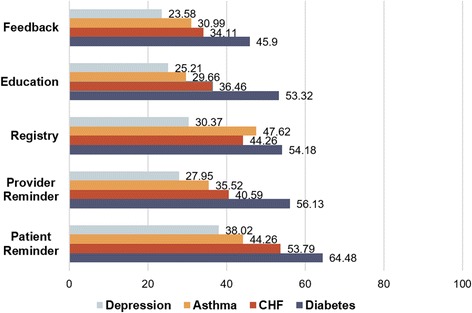
Adoption frequencies for care management practices, grouped by CMP type

Of the 1263 physician organizations with at least one CMP adopted, adoption of individual CMPs varied (see Fig. 2); feedback for depression was the least adopted CMP, adopted by less than one quarter (23.6%) of the organizations, and patient reminder for diabetes was the most adopted CMP, adopted by just under two thirds (64.5%) of the organizations. The five least adopted CMPs were all for depression, and the five most adopted CMPs were all for diabetes. Similarly, the feedback CMPs are usually less adopted than other CMPs for the same disease, whereas patient reminders tend to be adopted more often compared to other CMPs for the same disease.

As shown in Table 1, correlation coefficients for all 20 CMPs were positive and statistically significant (*p* ≤ 0.01). The correlation coefficients ranged from 0.21 to 0.95, with an average of 0.50. Within CMP pairs sharing the same disease focus, the average correlation coefficient was higher than the overall average at 0.55. Disease concordant CMP pairs with education as one of the CMPs tended to have correlations in the low range, whereas all other disease concordant CMP pairs had mostly moderate correlations. CMP pairs of the same type (e.g., both education or both registries) had even higher correlations than disease-focused pairs, with an average correlation coefficient of 0.88. The highest value for any correlation coefficient in the matrix was for asthma in the matrix was between feedback for asthma and for diabetes, *ρ* = 0.95, a very high correlation. The range of correlation values in the CMP type concordant group was high to very high. These findings suggest the Mokken scale analysis is appropriate in our pre-specified groupings.

**Table 1.**
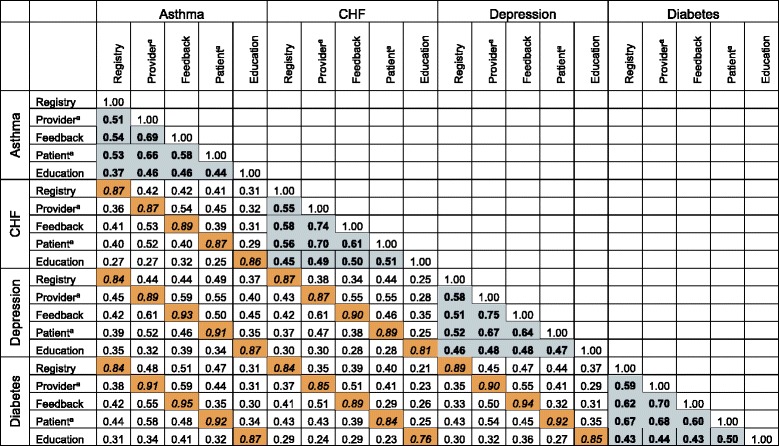
Care management practice tetrachoric correlation results

^a^Reminder: Cells with bolded text and light blue background are correlations between CMPs for the same disease; cells with italicized text and orange background are correlations between CMPs for the same CMP type; all correlation coefficients are statistically significant at the 0.01 level or lower

### Scale analyses

#### All-inclusive scale

**Fig. 3:**
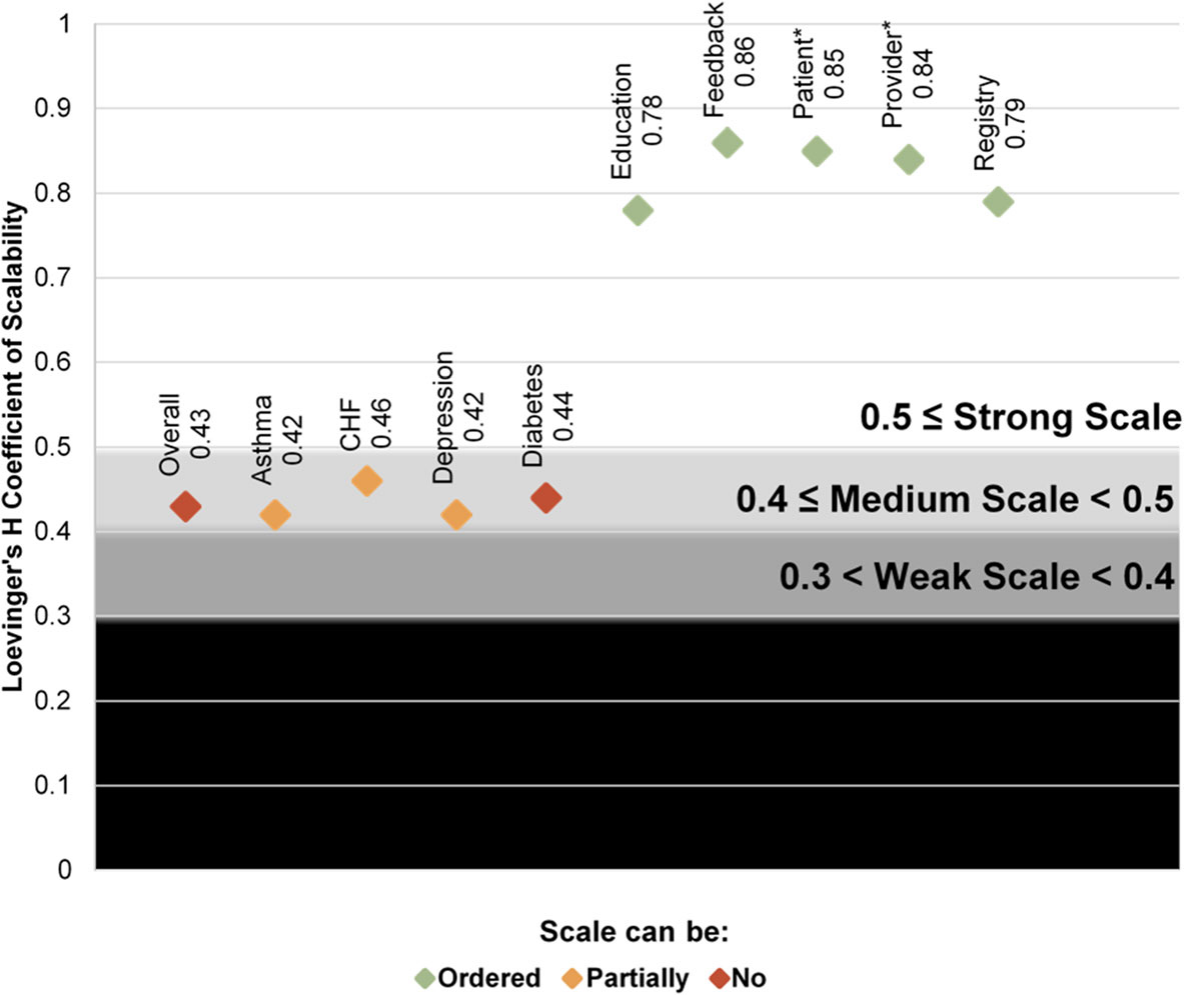
Mokken scale analysis results for scalability. Legend: asterisks indicate reminders

The Mokken scale for all 20 CMPs had medium scalability (Loevinger’s *H* coefficient of scalability = 0.43, see Fig. 3). This scale did not meet the additional assumption necessary for ranking CMPs, with all criteria values above the threshold value of 80 (see Additional file 1 for discussion of inclusion criteria and strength of scalability). Thus, this medium scale was detecting a latent trait shared by all 20 CMPs, but a consistent ranking or ordering of CMPs did not emerge.

#### Disease-focused scales

As shown in Fig. 3, scales for subsets of CMPs sharing a disease focus all had medium scalability (Loevinger’s *H* coefficient of scalability between 0.4 and 0.5 for all). However, in all scales, at least two CMPs had borderline values on inclusion criteria (i.e., values between 40 and 80), making it less clear if the assumption for ranking was met (see Additional file 1). Within the diabetes scale, education, provider reminders, and registries had high values, indicating lack of ordering of CMPs for diabetes. For the asthma, CHF, and depression scales, feedback and education had borderline criteria values, potentially demonstrating a lack of ordering for these two CMPs.

**Fig. 4:**
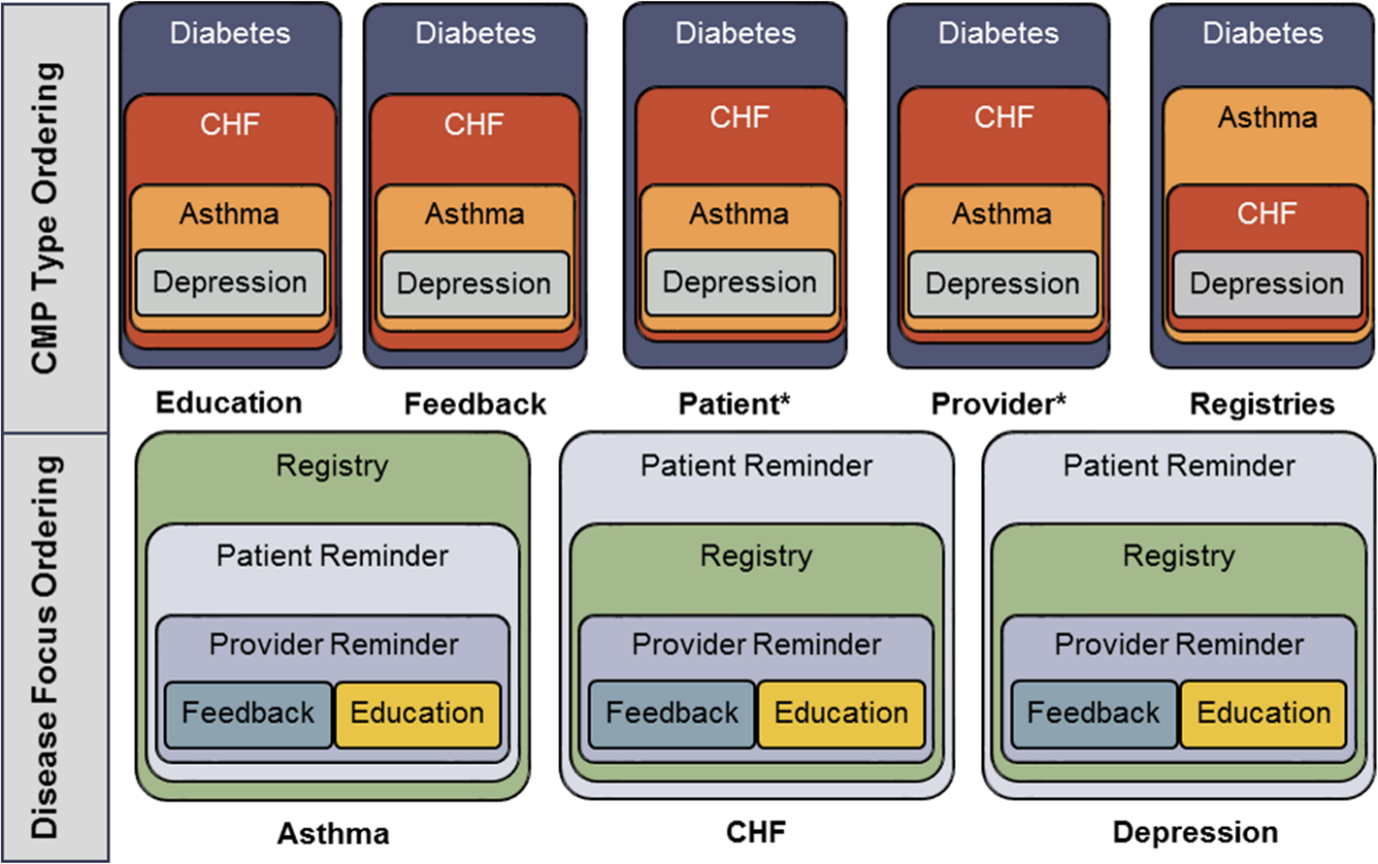
Ordering within disease focus and CMP type scales. Legend: asterisks indicate reminders note: Diabetes scale and overarching scales are not in the figure given their lack of ordering present. Each scale’s most adoptable practices is in the largest box and the least adoptable in the smallest. For disease-focused scales, feedback and education were both the least adoptable practices when run in separate scales and thus have equal rankings.

When the scales for asthma, CHF, and depression were tested with *either* feedback or education CMPs included in the analyses, the scales retained medium scalability, and the ranking assumption was met. This result confirmed that the other CMPs (education, provider reminders, patient reminders, and registries) within these scales were appropriate for ranking and ordering. Figure 4 depicts the ordering for all scales that could be ordered (excluding the diabetes scale and the overall scale, given their high inclusion criteria values). For asthma, the disease registries were the most adoptable CMP, followed by patient reminders and then provider reminders. For CHF and depression, patient reminders was most adoptable, followed by registry and then provider reminders. For all three disease scales, patient education ranked least adoptable when included, as did feedback when the patient education CMP was replaced by the provider feedback CMP.

As shown in Fig. 4, scales also indicate that organizations adopting provider reminders for asthma, CHF, or depression have typically also adopted patient reminders and registries for that same condition. For these conditions, all of these CMPs (provider reminders, patient reminders, and registries) will have been adopted before either education or feedback is adopted.

#### Shared CMP type scales

All scales including CMPs of the same type had strong scalability (Loevinger’s *H* coefficient of scalability > 0.5 for all, see Fig. 3) and satisfied the additional assumption allowing for ranking of CMPs within these scales (see Additional file 1). For education, feedback, patient reminder, and provider reminder CMP groups, diabetes was the most adoptable CMP, followed by CHF, then asthma, and finally depression (see Fig. 4). The one exception was for the registry CMPs, which ordered asthma as more adoptable than CHF.

These scales suggest that organizations that have adopted depression patient education have also adopted patient education for asthma, CHF, and diabetes (see Fig. 4). Similarly, the ordering of items within the patient education scale suggests that organizations adopting diabetes patient education will not typically have adopted patient education for any other disease. However, the overall scale including all 20 CMPs did not meet the assumption necessary for ranking (Additional file 1). These results indicate that organizations are not finishing one complete scale of CMPs before starting another scale, e.g., they may start one scale, then begin a second or third without finishing the first. For instance, an organization may initiate four of the five CMP type scales together, e.g., by adopting the two most adoptable CMPs from the patient reminder scale—patient reminders for diabetes and CHF patient reminders—and also provider reminders, a registry, and education for diabetes, rather than adopting all patient reminders before adopting other CMP types or adopting all diabetes CMPs before adopting the CHF CMP.

## Discussion

To our knowledge, our study is the first to examine empirically the ordering and patterns of CMP adoption choices within physician organizations. Our findings suggest that adoption choices are linked and that when faced with multiple adoption choices, specific innovation characteristics may matter to the adoption decision [12, 19]. So, while all CMPs share a common latent trait, characteristics such as disease focus and CMP type may matter in determining whether a CMP is considered more or less adoptable.

In terms of disease focus within CMP type scales, diabetes CMPs consistently ranked as the most adoptable and depression CMPs consistently emerged as the least adoptable. For rankings of CMP types within disease-focused scales, patient reminders and registries were found to be more adoptable, while feedback and education were identified as less adoptable. Provider reminders consistently fell third in ordering. Below, we review the relevance and practical implications from each set of scale analyses, beginning with the all-inclusive scale of CMPs, then discussing the disease focus scales, and finally describing the CMP type scales. We also describe potential extensions of this exploratory work and the questions it raises.

CMPs have shared conceptual links in models describing healthcare redesign for chronic care [13, 15], and the Mokken scale results support this overarching connection, having produced medium scalability for the full set of 20 CMPs, rather than breaking the scale into smaller subscales. That said, there was no ordering when considering all 20 CMPs within the all-inclusive scale. A ranking of the overall scale would imply that there was only one order of adoptability detected across the entire sample of physician organization, which is highly unlikely in a natural diffusion scenario. This warrants further exploration to better understand how the subscales, which do have ordering, relate to one another. It might be that different sets of CMPs are adopted in parallel, with physician organizations either tackling multiple scales simultaneously (e.g., starting both the CHF and asthma subscales) or pausing one subscale to begin another.

For disease-focused scales, scalability was also medium and the ordering of CMPs was possible, with some caveats. Within CMP type scales, diabetes was the most adoptable CMP, followed by either CHF or asthma; depression was consistently the least adoptable CMP. In mapping CMP characteristics to innovation traits described in the Diffusion of Innovations literature, several potential explanations for the adoption ordering seem plausible. First, the high prevalence and cost associated with diabetes compared to the other three chronic diseases [22–26] suggests that physician organizations may consider the fit with organizational priorities when deciding which CMPs to adopt. However, disease prevalence alone may not be sufficient to drive the adoption decision. It is possible that physician organizations’ experiences with adopting diabetes CMPs may shape their ordering preferences for subsequent CMP adoption. Diabetes CMPs also have the distinction of an extensive evidence base [13, 15], which may make diabetes CMP adoption more attractive, because the relative advantage of these CMPs is borne out in the evidence. In this study, depression CMPs were consistently identified as the least adoptable even though depression is more prevalent than CHF [25, 26], and there is a stronger evidence base for the effectiveness of depression CMPs than for CHF CMPs [36]. This discrepancy may be attributed to lower innovation-task fit of depression in primary care, as physician organizations struggle to realign primary care professionals’ roles to incorporate behavioral and mental healthcare [37], or to normative pressures within primary care that limit primary care providers’ motivation for investing in and/or otherwise developing expertise in depression care [38–40]. Finally, findings indicated that diabetes was the only disease-focused scale in which CMPs were not adopted in a specific order.

When comparing the different CMP types, strategies like patient reminders and registries, the two most adoptable CMPs, appear to be less complex to implement compared to the others, as they require relatively less maintenance or investment from the physician organization once implemented. Provider reminders, patient education, and physician feedback, in contrast, incentivize providers to action. For CMP types such as patient education and provider feedback, the additional complexity of a human resource and/or interpersonal interaction component may make these CMPs not only less adoptable but more difficult to implement and sustain [36]. Unlike registries and automated reminders, which require high up-front costs to design and adopt, but are relatively low-cost to maintain, CMPs such as provider feedback require ongoing resource investment in the form of data analysis, management, and clinician time.

### Limitations

Our data source has some inherent limitations. First, because we used cross-sectional data, we were not able to conduct analyses focused on the time sequencing of adoption decisions longitudinally or capture exnovation [36]. In addition, survey data used in this study contained information on only five CMP types across four chronic conditions. While these CMPs and conditions were selected based on their prevalence in the general population, they do not represent a comprehensive array of adoption choices being made by organizations at the same time; future research could examine patterns of adoption across other types of innovations and disease conditions.

Future work is needed to integrate contextual or organizational factors. For instance, past work has shown that variable diffusion particularly impacts physician organizations serving populations with high levels of socioeconomic vulnerability, as these organizations tend to fall behind in adoption efforts [27]. There is also evidence that characteristics like organizational size are linked to CMP adoption [12]. These issues could be addressed using different analytic techniques, like differential item functioning. Like Mokken analysis, differential item functioning falls under the item response theory umbrella, but they have different goals: Mokken analyzes patterns within a set of responses to establish if scales and ordering exist [20], whereas differential item functioning uses regression and other analyses to determine if different types of respondents answer differently to existing scales [41]. The preliminary work in this paper establishes scales which could then be used in subsequent differential item-functioning analyses. Data are also based on reports of a single key informant (lead physician or administrator) within each organization. While this informant was identified as being the most appropriate individual to answer questions related to organizational structure and resources, reliance on a single respondent still poses some risk of measurement error. Finally, findings may not generalize to the physician organizations outside our sample, since respondents to this survey may have differences from physician organizations that did not participate.

### Implications

Better understanding of care delivery innovation patterns may allow for more effective strategic implementation and dissemination efforts that are customized based on the organization’s current progress and the ordering they are likely to follow. For example, in an organization with no care management practices looking to make initial investments, a good introduction would be to begin with diabetes-related CMPs. Once some CMPs have been adopted, dissemination efforts could seek to expand upon this progress by promoting adoption of CMPs in the same CMP type, following the disease order we observed: diabetes, CHF, asthma, and finally depression. Future work is needed to determine how organizational characteristics such as size, ownership, and specialty composition might impact these adoption patterns.

This empirical assessment does address what physician organizations experience as less adoptable care management practices, but more work is needed to better understand why “less adoptable” CMPs may pose challenges or barriers for organizations. It may be that CMPs like provider feedback and patient education are less adoptable because they require human resources, expertise, and greater interpersonal communication, or that they take more effort to sustain on an ongoing basis. With more comparative work looking at the relative advantages and challenges of the various CMPs, it would be possible to better understand why these adoption choices are being made and how the two characteristics in this study relate to the innovation characteristics described by Rogers’s Diffusion of Innovations framework and others [5, 7–10]. Because innovation characteristics are relative to the adopter, organizations may judge the same innovation differently based on contextual factors like patient population or other organizational needs. For instance, the characteristic of compatibility would likely be different for CHF CMPs if an organization had a predominantly pediatric population, compared to an organization with a large geriatric patient population. Thus, characteristics described by the Diffusion of Innovations framework are not fixed and universal, and their relationship to the traits of CMP type and disease focus requires further study.

As rapid innovation in healthcare continues, organizations will be faced with a steady stream of decisions to adopt innovations and evidence-based practices. Rather than viewing each of these choices in isolation, the reality of these environments suggests that these adoption decisions are not made wholly independent of one another. Findings from our study demonstrate that shared traits between care management practices may provide an ordering of adoption choices.

## Conclusions

Organizations are adopting CMPs in a consistent pattern: diabetes is ranked the most adoptable, and depression is ranked the least adoptable. When looking within CMPs sharing a disease focus, patient reminders are ranked the most adoptable and feedback and education are ranked as both being the least adoptable. Our study provides some of the first empirical evidence of ordering of adoption choices and builds from prior theory suggesting that characteristics of an innovation influence adoption decisions. A better understanding is needed of why certain CMPs are less adoptable, connecting these finding to the innovation characteristics described by the Diffusion of Innovations framework. The findings from this study may suggest which CMPs may be more adoptable for non-adopter organizations, while also describing a potential sequencing that future work could explore. More insight into how innovation characteristics factor into physician organizations’ adoption strategy could lead to more effective adoption of innovations and evidence-based practices.

## Additional file

Additional file 1: Description and results of specification tests. (DOCX 20 kb)

## Abbreviations

CHF: Congestive heart failure
CMP: Care management practice
