# Supplementary material for: Random or predictable?: Adoption patterns of chronic care management practices in physician organizations
Source: Implement Sci. 2017 Aug 24;12:106. doi: 10.1186/s13012-017-0639-z (PMC5571615; doi:10.1186/s13012-017-0639-z)
Supplement: Additional file 1: — Description and results of specification tests. (DOCX 20 kb) [file 13012_2017_639_MOESM1_ESM.docx]

ADDITIONAL FILE 1. Description and Results of Specification Tests

We first applied the monotonely homogeneous Mokken model (MHMM), which makes three assumptions about items within a scale: unidimensionality for a latent trait, local stochastic independence, and monotonicity [1, 2]. **Unidimensionality for a latent trait** refers to the assumption that all items in the scale measure the same latent trait, e.g., adoptability. **Local stochastic independence** refers to the assumption that the responses to items by the same respondent are only influenced by the latent trait, and not by any other characteristics of the respondent or items. **Monotocity** refers to the assumption that the probability of a positive response to the items in question increases as the latent trait increases (or at least does not decrease). In testing for these assumptions, we are able to determine if responses to a particular set of items are representing one shared trait, or a scale.

If a scale satisfies the assumptions for the MHMM, it can be described as having good scalability and the Loevinger’s H coefficient of scalability will be close to 1 [1]; scales at or above 0.5 are considered strong, at or above 0.4 are considered medium, and above 0.3 are considered weak [3, 4]. Loevinger’s H coefficient of scalability for each scale tested can be found in the “Table of Results from Mokken Scale Analysis Specification Tests” below in the column “Overall H.” The resulting scalability strength is described in the “Overall scalability” column. For example, the education scale had an overall Loevinger’s H coefficient of scalability of 0.78, and is thus considered a strong scale.

Loevinger’s H coefficient of scalability describes to what extent the items within a scale are measuring the same latent trait in a consistent way across respondents and are not being influenced by other traits. The Loevinger’s H coefficient of consistency of an item within a scale is also taken into account, and should also be judged as the overall scale coefficient is [1]. In addition, to satisfy the assumption of monotonicity for each scale, we conducted a visual inspection of the traces of the items in the scale to ensure that the trace lines are increasing linearly. We also checked the monotonicity assumption using indexed criterion of each scale, which should ideally be below 40 for each item, but may be acceptable under 80 [5]. Criteria means and ranges for each scale are presented in the table below in the “MHMM” column. For MHMM scales with criteria under 80, items are measuring a latent trait, and increase as this trait increases.

For scales with acceptable MHMM properties, we then determined if the scale also satisfied the additional assumption necessary to demonstrate consistent ranking of items for all respondents. If satisfied, these sets of items could then be described as the doubly monotonely homogenous model of Mokken (DMHMM), in which items were both linked by a latent trait and demonstrated invariant item ordering [4].

The non-intersection of the Pmatrix curves is a goodness-of-fit diagnostic that shows non-intersection of items, or invariant item ordering [4, 5]. Based on the probabilities of a positive response in the overall group, items are ordered or ranked in two matrices: one matrix containing all joint proportions of respondents giving a positive response to items (P+ +) and the other containing all joint proportions of respondents giving a negative response to items (P- -) [6]. As we move up the order, the P+ + matrix should steadily increase across the rows and columns, and the P - - matrix should steadily decrease across the row and columns. Non-intersection of the Pmatrix curves represents this phenomenon.

If scales meet this additional assumption, they can then be described as the DMHMM. The inclusion criterion calculated for DMHMM must also fall below 40 for each item in the scale ideally, but may be acceptable under 80, just as with the criterion used above for monotonicity [1]. Inclusion criteria falling between 40 and 80 were considered borderline in this study. For DMHMM scales with criteria under 80, items are ranked consistently. Criteria means and ranges for each scale are presented in the table below in the “DMHMM” column, and the determination of whether items can be ranked or not is described in the last column under the overall scalability for each scale. For all Mokken analyses, we used the pairwise option to retain as much information as possible.

Table of Results from Mokken Scale Analysis Specification Tests

|  | Scale subgroup | Overall H^a^  *Individual H^b^ range* | MHMM^c^ mean, range | DMHMM^c^ mean, range | Overall scalability  Ranking^d^ |
| --- | --- | --- | --- | --- | --- |
| Modality | Education | 0.78  *0.73, 0.85* | -24  -27, -21 | -24  -27, -21 | Strong  Yes |
|  | Feedback | 0.86  *0.80, 0.92* | -28  -31, -25 | -28  -31, -25 | Strong  Yes |
|  | Patient Reminder | 0.85  *0.81, 0.89* | -28  -30, -25 | -28  -30, -25 | Strong  Yes |
|  | Provider Reminder | 0.84  *0.79, 0.90* | -27  -30, -24 | -27  -30, -24 | Strong  Yes |
|  | Registry | 0.79  *0.75, 0.87* | -24  -28, -22 | -24  -28, -22 | Strong  Yes |
| Disease | Asthma | 0.42  *0.33, 0.45* | -5.8  -8, -2 | 15.2  -8, 51 | Medium  Maybe |
|  | CHF | 0.46  *0.37, 0.53* | -8  -12, -3 | 13.6  -12, 51 | Medium  Maybe |
|  | Depression | 0.42  *0.33, 0.47* | -6  -8, -2 | 16.8  -8, 55 | Medium  Maybe |
|  | Diabetes | 0.44  *0.33, 0.52* | -7.4  -11, -1 | 37.8  -11, 99 | Medium  No |
| Overall scale | | 0.43  *0.32, 0.56* | 0.9  -13, 31 | 194.05  100, 256 | Medium  No |

^a^Loevinger’s H coefficient of scalability; ^b^Individual Loevinger’s H coefficient measuring the consistency of an item within a scale; ^c^criteria scores for items within a MHMM scale: < 40 then scale acceptable, between 40 to 80 then scale may be acceptable, above 80 assumption is not met for scaling; ^d^criteria scores for items within a DMHMM scale: < 40 then ranking acceptable, between 40 to 80 then ranking may be acceptable, above 80 assumption is not met for ranking.

**REFERENCES FOR ADDITIONAL FILE 1**

1. Hardouin J-B, Bonnaud-Antignac A, Sébille V**.** Nonparametric item response theory using Stata. Stata Journal. 2011;11(1):30.

2. Van Schuur WH**.** Mokken scale analysis: between the Guttman scale and parametric item response theory. Political Analysis. 2003;11(2):139-63.

3. Mokken RJ**.** *A theory and procedure of scale analysis: With applications in political research*: Walter de Gruyter; 1971.

4. Mokken RJ, Lewis C**.** A nonparametric approach to the analysis of dichotomous item responses. Applied Psychological Measurement. 1982;6(4):417-30.

5. Molenaar IW, Sitjtsma K, Boer P**.** User's Manual for MSP5 for Windows: A Program for Mokken Scale Analysis for Polytomous Items (Version 5.0). Groningen, The Netherlands: University of Groningen; 2000.

6. Reise SP, Revicki DA**.** *Handbook of item response theory modeling: Applications to typical performance assessment*: Routledge; 2014.
